# Supplementary material for: CRISPR Editing Enables Consequential Tag-Activated MicroRNA-Mediated Endogene Deactivation
Source: Int J Mol Sci. 2022 Jan 19;23(3):1082. doi: 10.3390/ijms23031082 (PMC8834719; doi:10.3390/ijms23031082)
Supplement: Supplementary file 1 [file ijms-23-01082-s001.zip › ijms-1538827-supplementary.pdf]

Supplementary Material

CRISPR Editing Enables Consequential Tag-Activated MicroRNA-Mediated Endogene Deactivation

Panayiota L. Papasavva [1,2], Petros Patsali [1,2], Constantinos C. Loucari [1,2], Ryo Kurita [3], Yukio Nakamura [4], Marina Kleanthous [1,2,†] and Carsten W. Lederer [1,2,*,†]

1 Department of Molecular Genetics Thalassemia, The Cyprus Institute of Neurology and Genetics, Nicosia 2371, Cyprus; panayiotap@cing.ac.cy (P.L.P.); petrospa@cing.ac.cy (P.P.); loucari.constantinos@gmail.com (C.C.L.); marinakl@cing.ac.cy (M.K.)
2 Cyprus School of Molecular Medicine, Nicosia 2371, Cyprus
3 Research and Development Department, Central Blood Institute, Blood Service Headquarters, Japanese Red Cross Society, Koto-ku, Tokyo 135-8521, Japan; r-kurita@jrc.or.jp
4 Cell Engineering Division, RIKEN BioResource Research Center, Tsukuba 305-0074, Japan; yukio.nakamura@riken.jp
* Correspondence: lederer@cing.ac.cy
† Contributed equally to this work.

Table S1. Detected erythromiRs in CD34+ and HUDEP-2 cells sorted by late-erythroid expression

erythromiR	late-erythroid expression[1]	log_2 fold change (late vs early)[2]
**hsa-miR-451a**	30227.74	4.394422
**hsa-let-7a-5p**	18887.48	1.275848
**hsa-miR-16-5p**	18473.67	1.283366
**hsa-let-7b-5p**	8885.507	2.235738
**hsa-miR-22-3p**	5006.113	2.003479
**hsa-miR-15b-5p**	4237.653	2.705584
**hsa-miR-1246**	4041.64	1.336994
**hsa-miR-486-5p**	3326.803	1.642032
**hsa-miR-12136**	713.3783	1.945231
**hsa-miR-26b-5p**	498.4767	1.162958
**hsa-miR-15a-5p**	474.665	3.129503
**hsa-miR-182-5p**	463.9217	3.836165
**hsa-miR-183-5p**	245.3	3.86204
**hsa-miR-194-5p**	124.6483	1.744664
**hsa-miR-324-5p**	85.535	1.186376
**hsa-miR-4488**	60.32	2.472766
**hsa-miR-2110**	36.21167	2.465297
**hsa-miR-629-5p**	34.18667	2.605443
**hsa-miR-96-5p**	33.9	3.76696
**hsa-miR-589-5p**	26.34833	1.059306
**hsa-miR-320d**	20.59	1.941568
**hsa-miR-320c**	18.29667	2.08572
**hsa-miR-505-5p**	14.03667	4.395572
**hsa-miR-22-5p**	11.71667	3.776355
**hsa-miR-4732-3p**	11.20333	1.479488
**hsa-miR-1843**	7.275	1.627817
**hsa-miR-5010-3p**	6.801667	1.747507
**hsa-miR-5010-5p**	4.846667	4.618168
**hsa-miR-194-3p**	4.063333	1.714765
**hsa-miR-550b-3p**	3.81	1.159548
**hsa-miR-628-3p**	2.996667	1.908415

**hsa-miR-3688-3p**	2.328333	1.707055
**hsa-miR-4732-5p**	2.271667	2.883218
**hsa-miR-4516**	2.133333	2.131863
**hsa-miR-548at-5p**	1.661667	1.620527
**hsa-miR-1306-3p**	1.661667	1.217872
**hsa-miR-1255b-5p**	1.303333	1.093899
**hsa-miR-663b**	1.203333	2.058942
**hsa-miR-3189-3p**	1.186667	2.308404
**hsa-miR-6513-3p**	1.113333	4.453584
**hsa-miR-6842-5p**	1.075	2.944518
**hsa-miR-11401**	0.913333	1.181792
**hsa-miR-4422**	0.843333	1.406704
**hsa-miR-4492**	0.79	1.737284
**hsa-miR-616-3p**	0.68	2.021614
**hsa-miR-1225-5p**	0.656667	2.779737
**hsa-miR-133a-3p**	0.611833	2.139851
**hsa-miR-483-5p**	0.573333	4.218646
**hsa-miR-3140-3p**	0.565	1.681019
**hsa-miR-6741-5p**	0.556667	1.174868
**hsa-miR-3605-5p**	0.526667	1.429845
**hsa-miR-3688-5p**	0.491833	1.953919
**hsa-miR-1249-5p**	0.490167	1.310045
**hsa-miR-4449**	0.488333	1.255644
**hsa-miR-183-3p**	0.460167	3.121828
**hsa-miR-4747-5p**	0.388333	4.346226
**hsa-miR-3619-3p**	0.361833	2.460143
**hsa-miR-6884-5p**	0.356667	2.694188
**hsa-miR-3667-5p**	0.355	1.494749
**hsa-miR-4525**	0.3485	3.747597
**hsa-miR-6505-3p**	0.345	1.684867
**hsa-miR-6825-5p**	0.330167	6.509475
**hsa-miR-664b-5p**	0.305333	1.8195
**hsa-miR-3191-5p**	0.303333	2.856571
**hsa-miR-4429**	0.296667	3.505062
**hsa-miR-4489**	0.286833	1.541892
**hsa-miR-3190-5p**	0.275333	6.671596
**hsa-miR-5695**	0.271667	1.777872
**hsa-miR-184**	0.27	2.575449
**hsa-miR-3190-3p**	0.238333	2.466754
**hsa-miR-7155-3p**	0.226667	2.269027
**hsa-miR-6837-5p**	0.135167	3.199413
**hsa-miR-6800-5p**	0.123333	1.895501

[1]: mean normalized miRNA counts in late-erythroid samples and [2]: mean log_2 fold change of miRNA expression in late- vs early-erythroid samples, both as calculated by DEGseq [37]

Table S2. Synthetic, modified oligonucleotides used as donor DNA

Type	Oligo_Name	Annotation	Sequence (5’>3’)
**dsODNs donors for NHEJ-mediated delivery**	dsODN451-2MRSs (ordered as DNA duplex from Metabion)	Sense	5’-P-A*A*CTCAGTAATGGTAACGGTTTGGCCAACTCAGTAATGGTAACGGT*T*T
		Antisense	5’-P-A*A*ACCGTTACCATTACTGAGTTGGCCAAACCGTTACCATTACTGAG*T*T
	dsODN451-4MRSs (ordered as DNA duplex from Metabion)	Sense	5’-P-A*A*CTCAGTAATGGTAACGGTTTGGCCAACTCAGTAATGGTAACGGTTTCAT CAACTCAGTAATGGTAACGGTTTCGATAACTCAGTAATGGTAACGGT*T*T
		Antisense	5’-P-A*A*ACCGTTACCATTACTGAGTTATCGAAACCGTTACCATTACTGAGTTGATG AAACCGTTACCATTACTGAGTTGGCCAAACCGTTACCATTACTGAG*T*T
	dsODN451-2MRSs (ordered as DNA oligo from IDT)	Sense	5’-P-A*A*CTCAGTAATGGTAACGGTTTGGCCAACTCAGTAATGGTAACGGT*T*T
		Antisense	5’-P-A*A*ACCGTTACCATTACTGAGTTGGCCAAACCGTTACCATTACTGAG*T*T
	dsODN451-4MRSs (ordered as DNA oligo from IDT)	Sense	5’-P-A*A*CTCAGTAATGGTAACGGTTTGGCCAACTCAGTAATGGTAACGGTTTCAT CAACTCAGTAATGGTAACGGTTTCGATAACTCAGTAATGGTAACGGT*T*T
		Antisense	5’-P-A*A*ACCGTTACCATTACTGAGTTATCGAAACCGTTACCATTACTGAGTTGATG AAACCGTTACCATTACTGAGTTGGCCAAACCGTTACCATTACTGAG*T*T
	GUIDESeq-dsODN (ordered as DNA Oligo from IDT)	Sense	5’-P-G*T*TTAATTGAGTTGTCATATGTTAATAACGGT*A*T
	GUIDESeq-dsODN (ordered as DNA Oligo from IDT)	Antisense	5’-P-A*T*ACCGTTATTAACATATGACAACTCAATTAA*A*C
**ssODNs for HDR-mediated delivery**	ssODN451TS-1MRS	Ultramer DNA Oligo	A*A*G GGA CAA AAA GCA CAC TAC ATA ACA AAC CAA CGA AAC CGT TAC CAT TAC TGA GTT TTA TTA GCT TGC AGT ACT GCA TAC AGT ATG GCA* G*C
	ssODN451NTS-1MRS	Ultramer DNA Oligo	G*C*T GCC ATA CTG TAT GCA GTA CTG CAA GCT AAT AAA ACT CAG TAA TGG TAA CGG TTT CGT TGG TTT GTT ATG TAG TGT GCT TTT TGT CCC* T*T
	ssODN451TS-2MRSs	Ultramer DNA Oligo	A*A*G GGA CAA AAA GCA CAC TAC ATA ACA AAC CAA CGA AAC CGT TAC CAT TAC TGA GTT GGC CAA ACC GTT ACC ATT ACT GAG TTT TAT TAG CTT GCA GTA CTG CAT ACA GTA TGG CA*G* C
	ssODN451NTS-2MRSs	Ultramer DNA Oligo	G*C*T GCC ATA CTG TAT GCA GTA CTG CAA GCT AAT AAA ACT CAG TAA TGG TAA CGG TTT GGC CAA CTC AGT AAT GGT AAC GGT TTC GTT GGT TTG TTA TGT AGT GTG CTT TTT GTC CC*T* T
	Alt-R HDR-2MRSs	Alt-R HDR Donor Oligo	A*A*G GGA CAA AAA GCA CAC TAC ATA ACA AAC CAA CGA AAC CGT TAC CAT TAC TGA GTT GGC CAA ACC GTT ACC ATT ACT GAG TTT TAT TAG CTT GCA GTA CTG CAT ACA GTA TGG CA*G* C

*P denotes a 5′ phosphorylation and * indicates a phosphorothioate linkage.*

Table S3. Oligonucleotides for cloning into the gRNA scaffold of lentiCRISPRv2

Target site	Orientation	Oligonucleotides (5’>3’)
**SC**	Sense	CACCGGCTTGCGGCGAGACATGG
	Antisense	AAACCCATGTCTCGCCGCAAGCC
**3’ UTR 1**	Sense	CACCGGTACTGCAAGCTAATAACGT
	Antisense	AAACACGTTATTAGCTTGCAGTACC
**3’ UTR 2**	sense	CACCGAGTGTATTTAATTGCGTTCC
	antisense	AAACGGAACGCAATTAAATACACTC
**3’ UTR 3**	Sense	CACCGGTTTGCTCAGCAACGAATTA
	Antisense	AAACTAATTCGTTGCTGAGCAAACC
**3’ UTR 4**	Sense	CACCGTATGATTTATTAGCACAACG
	Antisense	AAACCGTTGTGCTAATAAATCATAC
**5’ UTR 1**	Sense	CACCGGACGACGGCTCGGTTCACAT
	Antisense	AAACATGTGAACCGAGCCGTCGTCC
**5’ UTR 2**	Sense	CACCGACGACGGCTCGGTTCACATC
	Antisense	AAACGATGTGAACCGAGCCGTCGTC
**5’ UTR 3**	Sense	CACCGGAGTCTCCTTCTTTCTAACC
	Antisense	AAACGGTTAGAAAGAAGGAGACTCC

Table S4. Primers

Name	Orientation	Sequence (5'>3')
**SC**	Forward	AAAGCCATGACGGCTCTCCCACAAT
	Reverse	CGGCAATGGTTCCAGATGGG
**3' UTR 1**	Forward	ATTTGGCAGGATAATATAGTGC
	Reverse	CGGGCTTTCTCTTTAATATG
**3' UTR 2**	Forward	TTGTATTTCTCACAACAATGGCTAC
	Reverse	CCTCTTCCTTTCAATATGGTATACA
**3' UTR 3**	Forward	CCTTTATGTTCTCACCGTTT
	Reverse	CCTCCTCACGTTATAAAATA
**3' UTR 4**	Forward	CTGGCACCAAAAGAAATAGA
	Reverse	TTTAGGGAGCACAGACATAT
**5' UTR 1**	Forward	AAGCCATGACGGCTCTCCCACAAT
	Reverse	CCTGCGCGCTCTCGTGATTA
**5' UTR 2**	Forward	AAGCCATGACGGCTCTCCCACAAT
	Reverse	CCTGCGCGCTCTCGTGATTA
**5' UTR 3**	Forward	AAGCCATGACGGCTCTCCCACAAT
	Reverse	CCTGCGCGCTCTCGTGATTA
**DONOR 451**	Forward	GGTTTGGCCAACTCAGTAATG
**lentiCRISPRv2**	Forward	GAGGGCCTATTTCCCATGATT

Table S5. Synthetic gRNAs for ribonucleoprotein complex formation

Target site	CRISPR RNA (crRNA) (5'>3')
**SC**	GCTTGCGGCGAGACATGG
**3' UTR 1**	GTACTGCAAGCTAATAACGT
**3'UTR 4**	TATGATTTATTAGCACAACG
**5' UTR 1**	GACGACGGCTCGGTTCACAT
**5' UTR 3**	GAGTCTCCTTCTTTCTAACC

Table S6. Antibodies used in immunoblotting

Type	Antibodies	Dilution	Diluent
**Primary antibodies**	Mouse Anti-Ctip1/BCL11A antibody; ab19487	1:1,000	1% BSA/1× TBS-0.05% Tween 20
	Hemoglobin α (H-80) rabbit polyclonal IgG; sc-21005	1:1,000	1% BSA/1× TBS-0.05% Tween 20
	Hemoglobin γ (51-7) mouse monoclonal IgG1; sc-21756	1:1,000	1% BSA/1× TBS-0.05% Tween 20
	Anti-β-actin (AC-15) mouse monoclonal IgG1; A-1978	1:10,000	1% skimmed milk/1× TBS-0.05% Tween 20
**Secondary antibodies**	Peroxidase-conjugated AffiniPure goat anti-mouse IgG (H+L); 115-035-003	1:8,000	3% skimmed milk/1× TBS-0.05% Tween 20
	Peroxidase-conjugated AffiniPure goat anti-rabbit IgG (H+L); 111-035-003		

TBS: Tris-buffered saline, Tween 20: TWEEN® 20 Detergent from Sigma-Aldrich, Munich, Germany

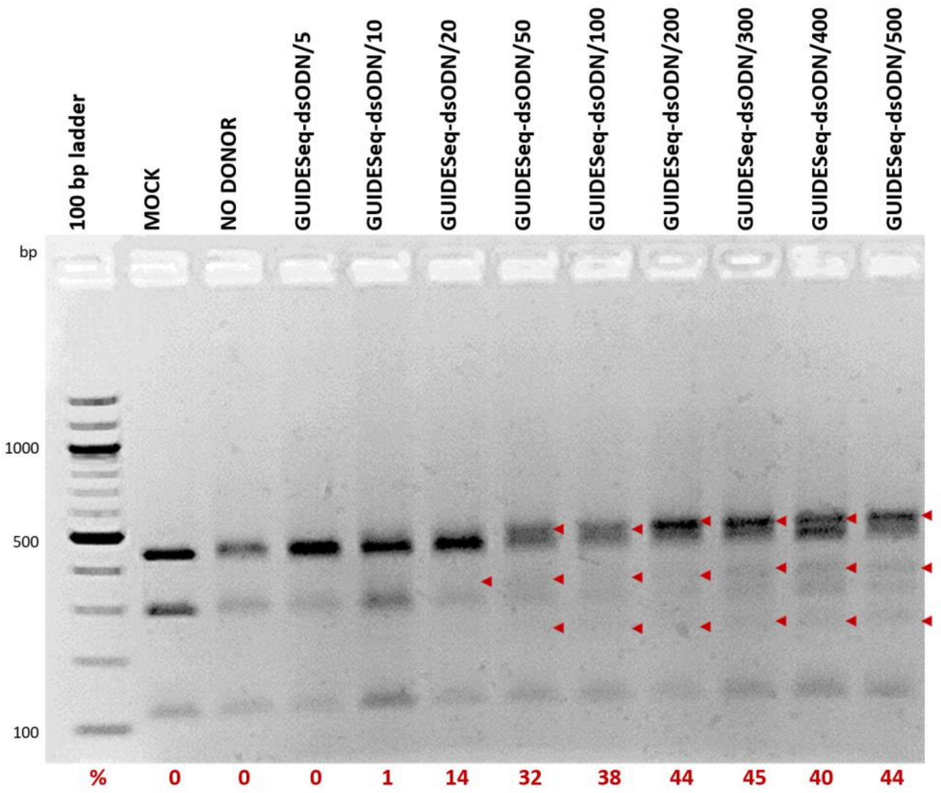

Figure S1. NHEJ-mediated integration of GUIDESeq-dsODN in HEK293T cells. PCR-RFLP analysis of HEK93T cells nucleofected with RNPs and GUIDESeq dsODN was performed to assess integration efficiency. Bands of 436, 288 and 118 bp represent absence of insertion, bands larger than 436 bp represent insertions with incomplete digestion by DdeI, and bands of 323 and 234 bp represent cleavage products of insertions. All bands corresponding to insertions are indicated by arrowheads. Corresponding rates of Dde cleavage (%) (after subtraction of background average cleavage rate of control samples) are reported below the gel. MOCK: mock-nucleofected sample, NO DONOR: cell sample nucleofected only with RNPs, GUIDESeq-dsODN/*: cell samples nucleofected with RNPs and GUIDESeq-dsODN at the indicated picomole quantity. The gel image was processed with an automatic noise filter to remove fluorescent inclusion artefacts.

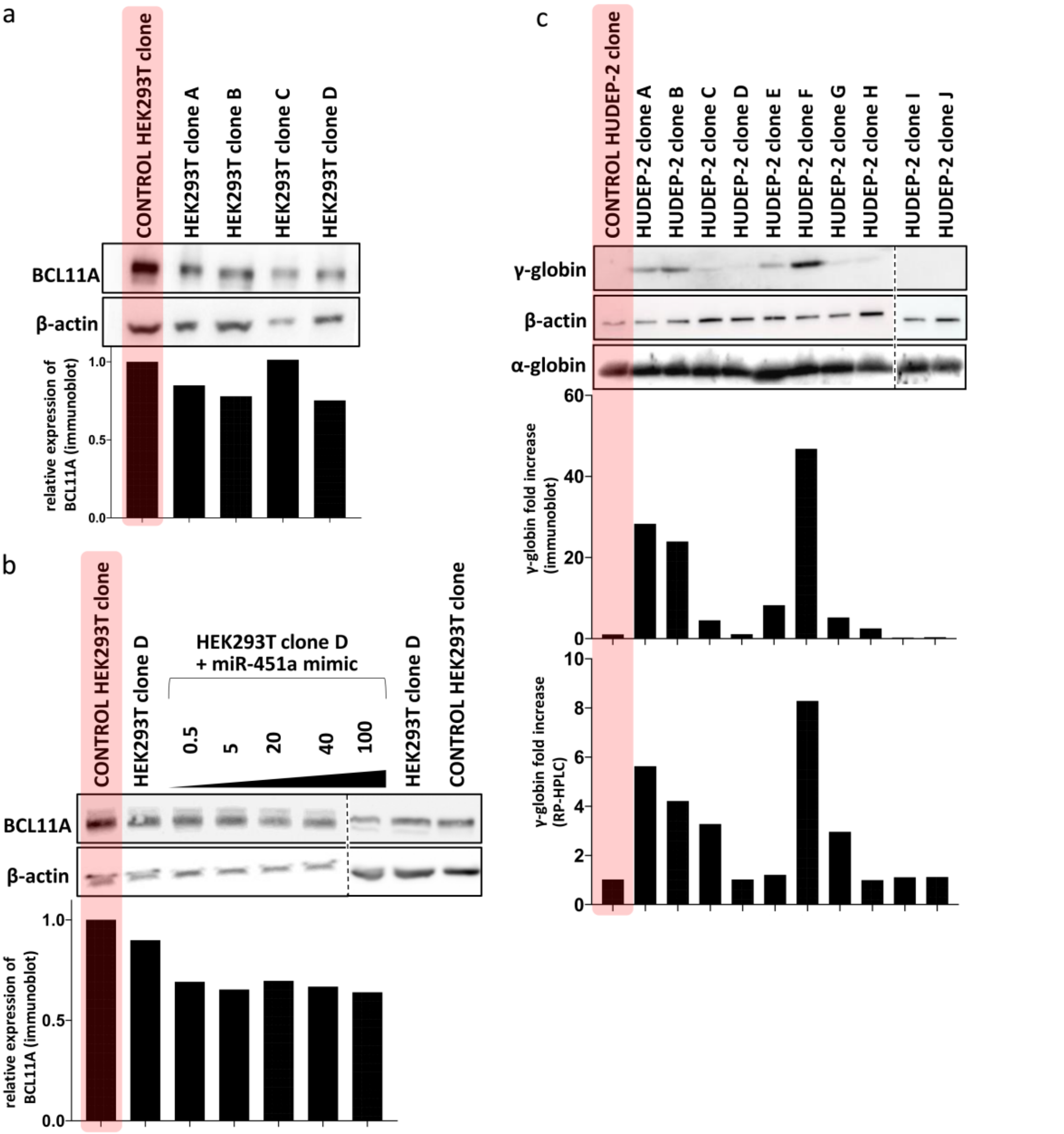

Figure S2. Analysis of monoclonal cell populations at the protein level. One sample per clone (n=1) was analyzed. (a) Immunoblot analysis of HEK293T clones bearing MRSs for miR-451a showed slightly diminished BCL11A expression in clones A (by ~15%), B (by ~22%) and D (by ~25%), relative to expression in a non-transfected wild-type CONTROL HEK293T clone. (b) Transient transfection of 0.5–100 pmole of synthetic hsa-miR-451a miRNA mimic in HEK293T clone D (clone harboring 4 MRSs in homozygosity) resulted in up to ~36% repression of BCL11A expression relative to expression in CONTROL HEK293T clone, and up to 26% compared to the non-transfected HEK293T clone D. (c) Immunoblot analysis of HUDEP-2 clones for γ-globin expression after erythroid differentiation showed 28.3-, 23.9- and 46.8-fold increase in HUDEP-2 clones A, B and F, respectively, and <6-fold increase in clones C, E, G, H, relative to expression in a non-transfected wild-type CONTROL HUDEP-2 clone. Expression of γ-globin was normalised to β-actin (for protein loading) and α-globin (for the level of erythroid differentiation), respectively. Less sensitive RP-HPLC analysis of the same clones showed 5.62-, 4.20- and 8.28-fold increase of γ-globin expression in clones A, B and F, respectively, and <3.5-fold increase in clones C and G. Control bands and values are designated with red overlays. Results for γ-globin expression showed good correlation between the two analysis methods ($r = 0.952$, $R^2 = 0.907$, $p < 0.0001$). Separate blots are indicated by dashed lines. CONTROL HEK293T clone: non-transfected wild-type HEK293T clone, HEK293T clone D: non-transfected HEK293T clone D, HEK293T clone D_miR-451a mimic: HEK293T clone D transfected with miR-451a miRNA mimic with quantities of 0.5–100 pmole.

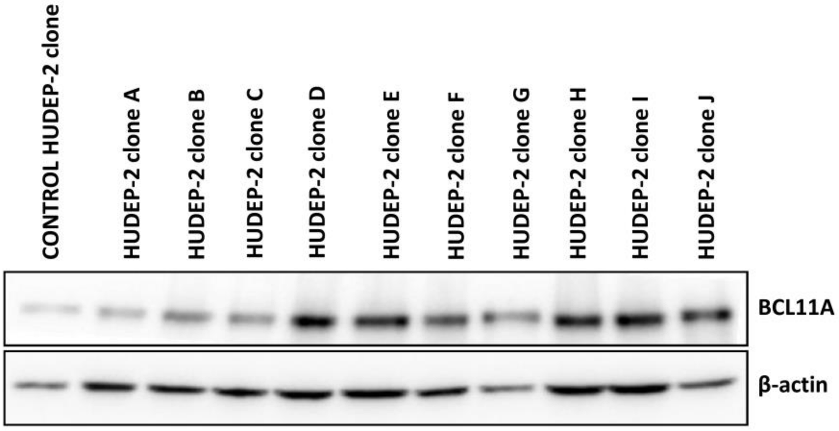

Figure S3. Immunoblot analysis of BCL11A expression in HUDEP-2 clones. The analysis was performed on D4 of erythroid differentiation (intermediate stage) and showed reduction of BCL11A expression only in HUDEP-2 clone A (27% relative to the wild-type CONTROL HUDEP-2 clone) and variably also upregulation in few others (up to 183% for HUDEP-2 clone J).
